# Hemokinin-1 is a mediator of chronic restraint stress-induced pain

**DOI:** 10.1038/s41598-023-46402-7

**Published:** 2023-11-16

**Authors:** Éva Borbély, Angéla Kecskés, József Kun, Eszter Kepe, Barbara Fülöp, Katalin Kovács-Rozmer, Bálint Scheich, Éva Renner, Miklós Palkovits, Zsuzsanna Helyes

**Affiliations:** 1 Department of Pharmacology and Pharmacotherapy, Medical School, University of Pécs, Pécs, Hungary; 2 Centre for Neuroscience, University of Pécs, Pécs, Hungary; 3 Department of Pathology and Experimental Cancer Research, Faculty of Medicine, Semmelweis University, Budapest, Hungary; 4 Human Brain Tissue Bank, Semmelweis University, Budapest, Hungary; 5 PharmInVivo Ltd, Pécs, Hungary; 6 Chronic Pain Research Group, Hungarian Research Network, University of Pécs, Pécs, Hungary; 7 National Laboratory for Drug Research and Development, Magyar Tudósok Krt. 2, Budapest, 1117 Hungary; 8 Department of Pharmaceutical Chemistry, Faculty of Pharmacy, University of Pécs, Pécs, Hungary

**Keywords:** Stress and resilience, Neuroimmunology

## Abstract

The *Tac4* gene-derived hemokinin-1 (HK-1) binds to the NK1 receptor, similarly to Substance P, and plays a role in acute stress reactions and pain transmission in mice. Here we investigated Tac4 mRNA expression in stress and pain-related regions and its involvement in chronic restraint stress-evoked behavioral changes and pain using *Tac4* gene-deleted (*Tac4*^-/-^) mice compared to C57Bl/6 wildtypes (WT). Tac4 mRNA was detected by in situ hybridization RNAscope technique. Touch sensitivity was assessed by esthesiometry, cold tolerance by paw withdrawal latency from 0°C water. Anxiety was evaluated in the light–dark box (LDB) and open field test (OFT), depression-like behavior in the tail suspension test (TST). Adrenal and thymus weights were measured at the end of the experiment. We found abundant Tac4 expression in the hypothalamic–pituitary–adrenal axis, but Tac4 mRNA was also detected in the hippocampus, amygdala, somatosensory and piriform cortices in mice, and in the frontal regions and the amygdala in humans. In *Tac4*^-/-^ mice of both sexes, stress-induced mechanical, but not cold hyperalgesia was significantly decreased compared to WTs. Stress-induced behavioral alterations were mild or absent in male WT animals, while significant changes of these parameters could be detected in females. Thymus weight decrease can be observed in both sexes. Higher baseline anxiety and depression-like behaviors were detected in male but not in female HK-1-deficient mice, highlighting the importance of investigating both sexes in preclinical studies. We provided the first evidence for the potent nociceptive and stress regulating effects of HK-1 in chronic restraint stress paradigm. Identification of its targets might open new perspectives for therapy of stress-induced pain.

## Introduction

Stress-induced chronic pain is a highly prevalent psychosocial problem worldwide^1–3^. Fibromyalgia patients experience long-lasting widespread pain, fatigue, as well as sleep disturbances and cognitive problems without any known underlying diseases. Besides chronic psychosocial stress, genetic, epigenetic, physical, and a broad range of environmental factors are involved in triggering the disease demonstrating its very complex pathophysiological mechanism^4^. The effectivity of the currently available pharmacotherapy is very poor: the approved analgesics alleviate pain by 50% only in 10–25% of the patients^5,6^. Moreover, their chronic use leads to wide range of adverse effects. Therefore, it is important to find new therapeutic targets for both chronic stress and pain to ensure better quality of life for these patients.

Pain- and stress-processing pathways are greatly overlapping^7,8^ (amygdala^9^, hippocampus, hypothalamus, periaqueductal gray matter^10,11^), and several mediators (glutamate; γ-aminobutyric acid, GABA^12^; neuropeptides, eg. tachykinins^13^) play regulatory roles in both systems. Since, we showed earlier that inhibition of the capsaicin-sensitive peripheral sensory neural system leads to aggravation of chronic restraint stress-induced pain in a mouse model, changes in neuroplasticity related to central sensitization in both stress- and pain-related brain regions are suggested to be involved^14,15^.

The newest tachykinin, hemokinin-1 (HK-1) encoded by the *Tac4* gene in mouse, share functional and immunological similarity to substance P (SP), encoded by *Tac1* gene. HK-1 was shown throughout the body, qPCR results revealed its expression in the lung, uterus, thymus and adrenals, while *Tac1* mRNA was mainly found in the capsaicin-sensitive sensory neural system and the brain^16^. Although HK-1 and SP share several common features and functions mainly by activating the NK1 receptor, they exert many effects related to pain and inflammation, due to distinct receptor and signaling pathways^17^. A broad range of results suggests that HK-1 is a potential regulator of several nervous system functions, but its role in stress and pain needs to be elucidated^18^. We provided evidence that HK-1 mediates hyperalgesia in pain states of neuropathic and inflammatory origin^19–21^, partially via NK1 receptor activation, but it also alleviates acute stress and depression-like behaviors independently of NK1 receptors in mouse models^22^. Human data also demonstrated the potential involvement of HK-1 in stress-pain interactions, since increased serum HK-1 level were measured in patients with fibromyalgia^15,23^.

Therefore, in this study we aimed to elucidate the presence of HK-1-encoding *Tac4* mRNA in stress- and pain-related brain regions, spinal cord, sensory neurons of trigeminal and dorsal root ganglia (TG, DRG), as well as in the hypothalamic–pituitary–adrenal (HPA) axis, and thymus as main regulators of stress responses. We used ultrasensitive RNAscope in situ hybridization technology to identify the *Tac4* mRNA-expressing mouse brain regions. For comparison, distinct human brain cortical areas and regions were also examined for human *Tac4* mRNA with RT-qPCR. Since HK-1 and SP share high sequence homology at protein level^24^, no specific monoclonal anti-HK-1 antibody is available commercially that could be used without potential cross reactivity with other tachykinins. Furthermore, we determined HK-1 function in chronic immobilization stress-induced hyperalgesia and behavioral changes in *Tac4* mice compared to NK1 receptor knockout onces. Since it is well-known that sex-dependent mechanisms influence both pain processing^25,26^ and mood regulation^27,28^, we investigated both male and female *Tac4* gene-deficient animals.

## Materials and methods

### Animals

Mice were housed in temperature and humidity controlled 12 h light–dark cycle environment in standard polycarbonate cages at the animal facility of the Department of Pharmacology and Pharmacotherapy University of Pécs. Mice were provided ad libitum with standard rodent chaw and drinking water. All procedures applied in this protocol were approved by the Ethical Committee on Use of Laboratory Animals at the University of Pécs (permission No: BAI/35/51–140/2016, BA/73/00,838–5/2021) and were performed according to the European legislation (Directive 2010/63/EU) and Hungarian Government regulation (40/2013., II. 14.) on the protection of animals used for scientific purposes. All in vivo experiments were complied with the ARRIVE guidelines.

All behavioral experiments were carried out on male and female 2–3-month-old *Tac4* gene-deficient (*Tac4*^*−/−*^) mice and C57Bl/6 J wildtypes. For comparison we used NK1 receptor knockout (*Tacr1*^*−/−*^) animals. The original breeding pairs of the *Tac4*^*−/−*^ mice were donated by Christopher J. Paige, Toronto, USA^29^, *Tacr1*^*−/−*^ mice were obtained from the University of Liverpool, UK^30,31^. Animals were bred on C57Bl/6 J background and backcrossed to homozygosity for > 5 generations prior to using C57Bl/6 J mice as controls (Charles-River Ltd., Hungary). Offspring was genotyped for *Tac4* and *Tacr1* gene by PCR. Animal number in the different experimental groups were: C57Bl/6 non-stressed male N = 12; C57Bl/6 stressed male N = 14; C57Bl/6 non-stressed female N = 8; C57Bl/6 stressed female N = 8; *Tac4*^*−/−*^ non-stressed male N = 6; *Tac4*^*−/−*^ stressed male N = 8; *Tac4*^*−/−*^ non-stressed female N = 8; *Tac4*^*−/−*^ stressed female N = 9; *Tacr1*^*−/−*^ non-stressed male N = 6; *Tacr1*^*−/−*^ stressed male N = 8.

### Post-mortem and neurosurgical human brain tissue samples

Different brain tissues were obtained for the RT-qPCR measurements from patients (N = 4–8) in short post-mortem delay (1–10 h) without any major neuropathological alterations. Tissue samples were microdissected in the Human Brain Tissue Bank, Semmelweis University. The human brain microdissection was approved by the Medical Research Council (ETT TUKEB); 5912–2/2018/EKU (Human Brain Tissue Bank, Semmelweis University, Budapest, Hungary), conducted in accordance with European directives and regulations. Medical history of the patients has been published previously^32^. Informed consent was obtained from all participants and/or their legal guardians.

### Characterizing *Tac4*-expressing neurons in the mouse nervous system by RNAscope in situ hybridization (RNAscope)

RNAscope was performed on 3-month-old male WT mice (N = 2). Briefly, animals were deeply anesthetized with an overdose of urethan (2.4 g/kg) and perfused transcardially with 4% paraformaldehyde in Millonig’s phosphate buffer (4% PFA). Dissected brains were postfixed for 48 h at 4 °C and sectioned (by 30 µm) using a vibrating microtome (VT1000S, Leica Biosystems, Wetzlar, Germany), then stored in 1 × PBS with 0.01% Na-azide until further use (Merck KGaA, Darmstadt, Germany). Dissected trigeminal ganglia (TG), lumbar spinal cords and pituitary glands were postfixed for 24h at 4 °C, rinsed in 1 × PBS, dehydrated and embedded in paraffin using standard procedures, then 5 µm sections were cut using a sliding microtome (HM 430, Thermo Fisher Scientific, USA). Dorsal root ganglia (DRG) were dissected and postfixed for 24h at 4 °C, cryoprotected in 30% sucrose in 10% neutral buffered formalin (Merck KGaA, Darmstadt, Germany) for 24 h at 4 °C and frozen in tissue freezing media (Leica Biosystems, Wetzlar, Germany) on dry ice. 20 µm sections were cut using cryostat (CM1850, Leica Biosystems). RNAscope assay was performed using RNAscope Multiplex Fluorescent Reagent Kit v2 (Advanced Cell Diagnostics, Newark, CA, USA) according to the manufacturer’s protocols with minor modifications as it was described earlier^33^. Sections were hybridized with probes specific to mouse *Tac4* (ACD, Cat. No. 449651-C2), *Vglut1* (ACD, Cat. No. 416631), *Vglut2* (ACD, Cat. No. 319171-C3), *Gad1* (ACD, Cat No. 400951-C3), *Crh* (ACD, Cat. No. 316091), *Calca* (ACD, Cat. No. 417961-C3) and *NeuN* mRNA (ACD, Cat. No. 313311-C3) in parallel with RNAscope 3-plex mouse positive (*Polr2a*, *Ppib*, *Ubc*, ACD, Cat. No. 320881) and negative control probes (ACD, Cat. No. 320871). In the case of RMg sections the *Tac4* and *Vglut2* RNAscope was combined with immunofluorescence of 5-hydroxytryptamine (5-HT). After the RNAscope procedure, slides were treated with monoclonal mouse anti-serotonin serum (gift from Dr. Lucienne Léger, Université Claude Bernard, Lyon, France) diluted 1:20,000. Superclonal Alexa Fluor 488 goat anti mouse IgG (Invitrogen Antibodies, Cat. No. A28175, 1:1000) was used as a secondary antibody. Sections were counterstained with DAPI and mounted with ProLong Glass Antifade Mountant (Thermo Fisher Scientific, Waltham, MA, USA) for confocal imaging. Samples were imaged by using LSM 710 confocal laser scanning microscope (Carl Zeiss, Jena, Germany). Virtual colors were selected to depict fluorescent signals: blue for DAPI, green for *Vglut1* (FITC), *Polr2a* (FITC), 5-HT (pseudo-colored), *Vglut2* (Cyanine 5, pseudo-colored), *Chr* (Cyanine 5, pseudo-colored), red for *Tac4* (Cyanine 3), white for *Gad1*, *Calca*, *NeuN* and *Ubc* (Cyanine 5). Brightness/contrast adjustment and z-projection (12–15 stacks/image, 2 µm-intervals) with maximum intensity of separate channels were processed using (Fiji, 1.53c, NIH, USA).

### Measuring *TAC4* levels in human brain samples with real-time (RT)-qPCR

*TAC4* mRNA levels were measured as described earlier^32^ Briefly, brain samples were homogenized (T 25 digital Ultra-TURRAX, IKA-Werke, Staufen, Germany) and total RNA extracted. The qPCR experiments were performed by Stratagene Mx3000P QPCR System with SensiFAST SYBR Lo-ROX Kit (Meridian Bioscience, Cincinnati, OH, USA). All qPCR measurements were executed in technical replicates. The geometric mean of the reference gene (importin 8 (*IPO8*), pescadillo homolog (*PES1*), DNA-directed RNA polymerase II subunit RPB1 (*POLR2A*) Ct values were calculated, and the relative human *TAC4* mRNA expression compared to the reference genes was determined by the 2^−ΔCt^ formula to compare distinct brain regions.

Detailed list of patient characteristics (age, gender, post-mortem delay, cause of death) as well as all primers are indicated in our previous publication^32^ and in Supplementary Table 1.

### Chronic restraint stress (CRS) paradigm

Stress was evoked by chronic restraint of the mice. Animals were placed into 50 mL plastic tubes, which holes for the appropriate ventilation, every day during the 4-week-long experimental period. The stress started in the morning and lasted for 6 h. Control, non-stressed, animals were handled identically, but not exposed to stress, they were left in their home cages for this period^14,34^.

Control measurements were carried out prior to the experiment to determine the baseline mechano-nociceptive threshold and cold tolerance of the mice. During the experiment nociceptive measurements with dynamic plantar aesthesiometry and withdrawal latency from 0 °C icy water was measured every week, behavioral tests were performed once at the last week of the experiment (Supplementary Figure 1). All tests were performed in the afternoon, at least 2 h after the stress and only one measurement was carried out on one day. The researcher was not informed about the stress and gene state of the animals.

At the end of the experiment mice were anesthetized with ketamine and xylazine and perfused with 4% PFA 24 h after the last restraint stress. Thymus and adrenal weights were measured.

### Nociceptive tests

#### Dynamic plantar aesthesiometry (DPA)

Mechanical touch sensitivity of the hind paws was assessed by DPA (Ugo Basile 37,400; Comerio, Italy). Mice can move freely in plastic boxes placed on a metal mesh above the touch stimulator. After 20 min acclimatization, the mechanonociception was determined on the plantar surface by increasing force exerted by a thin metal filament (ramp: 5 s, max. force: 10 g,). The force at which the mice withdrew their hind paws was recorded by the device and designate as the mechano-nociceptive threshold^35^. Average of the three measured values was calculated and the thresholds of the two sides were averaged.

#### Cold tolerance

Cold sensitivity of the hind paws was assessed by measuring of the withdrawal latencies from 0 °C icy water^14^. Animals were held gently while their hind paws were submerged separately in the water for 180 s, maximally. Withdrawal latency was determined, and the two sides were averaged.

#### Behavioral tests

Open field test (OFT)

OFT test is based on the conflict that mice have to choose between the aversion of rodents to bright, open places (which is the center zone of the experimental area) and the exploration of a new environment. Mice could move freely in a brightly lit plastic box (40 cm × 40 cm) and the behavior of the animals was recorded by a video camera during the 5-min-long experimental period. The time spent in the center zone, the latency to first entry to the center zone are determined to assess the anxiety level of the animals^22^. Body elongation is also a sign of risk assessment of rodents in a stressful situation^36,37^, therefore the time spent in stretched position is also measured with EthoVision XT 11 Software (Noldus Information Technology, Netherlands).

#### Light–dark box test (LDB)

The LDB test is an additional method to assess anxiety, the paradigm is very similar to OFT (light-aversion vs. exploration of a novel environment). LDB tests were performed in a 40 cm × 40 cm × 40 cm (L × W × H) wooden box, that consists of two equal (open, lit and closed, dark) compartments. Between the two compartments there is small opening at the floor level. Mice were individually investigated, and the time spent in the lit compartment and the number of transitions were determined during the 5-min experimental period^38^.

#### Tail suspension test (TST)

TST is a well-established, commonly used test for measuring anxiety/ depression-like behavior in mice. The test shows the dominant coping strategy of the animal, which can escape-oriented movements or immobility. It can be used for the investigation of potential antidepressant agents^39^, or for the examination of the behavior of genetically modified animals^40^. Mice were suspended by their tail (50 cm above the floor) with the help of an adhesive tape. The time spent immobile was measured in the last 4 min of the 6-min experimental period^41^.

### Statistics

Results were expressed as the means ± SEM of the groups in case of in vivo measurements and n = 2 mice for RNAscope in situ hybridization. Normality (Shapiro–Wilk, Kolmogorov–Smirnov) tests were performed before analysis, mechanical hyperalgesia and cold allodynia baseline values were evaluated with *t* test, their change after stress with two-way analysis of variance (ANOVA) followed by Bonferroni’s post hoc test, behavioral data with two-way ANOVA followed by Fischer’s post hoc test using GraphPad Prism9 software. In all cases *p* < 0.05 was considered as statistically significant.

## Results

### *Tac4* mRNA was expressed in brain areas implicated in pain, mood and odor processing

**Figure 1:**
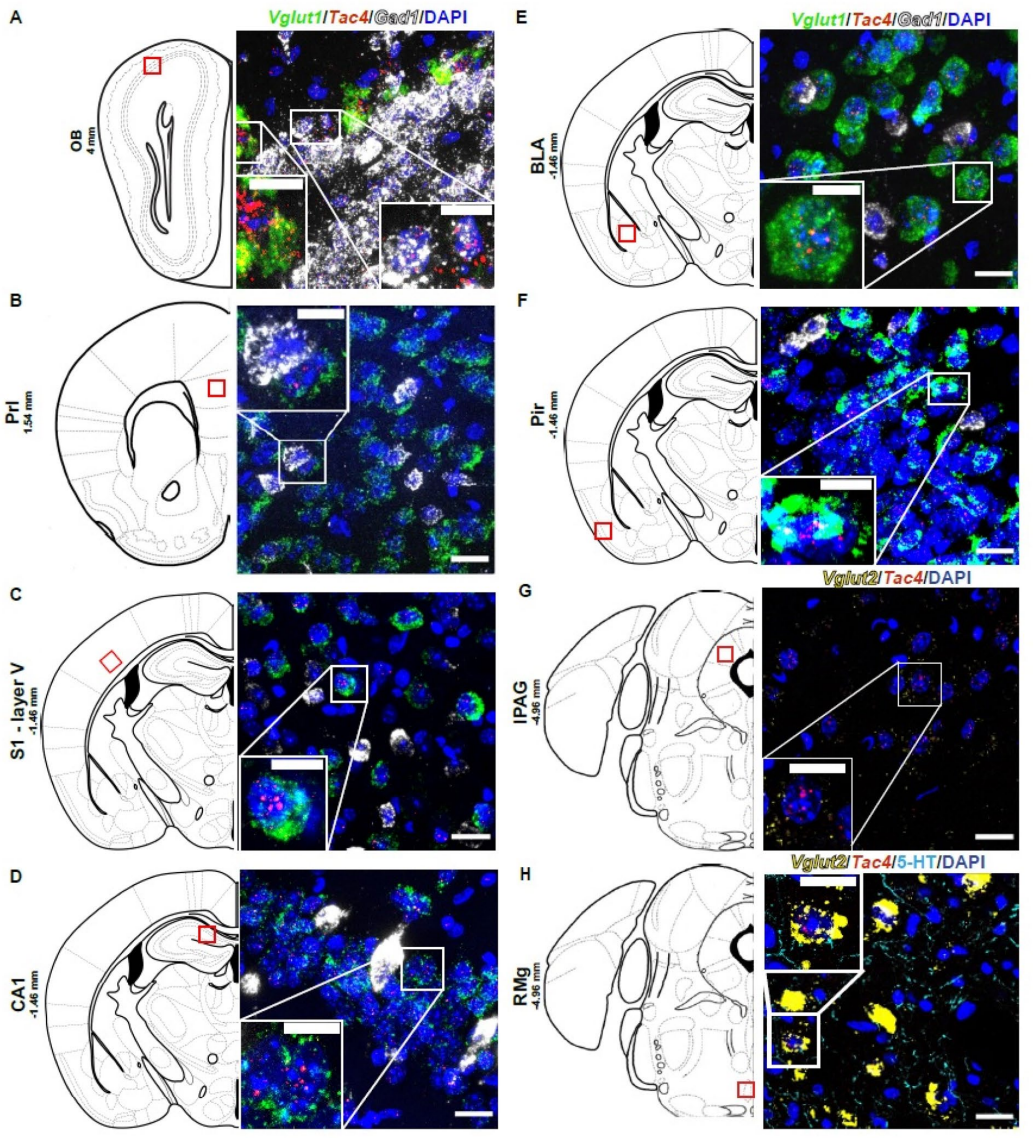
Mouse Tac4 mRNA primarily expressed by excitatory neurons. Representative confocal images of Tac4 mRNA (red) co-localized with Vglut1 (green) and Gad1 mRNA (white) in the olfactory bulb (OB, Bregma 4 mm, (**A**), in the prelimbic cortex (PrL, Bregma –1.54 mm, (**B**), in the primary somatosensory cortex (S1, Bregma –1.46 mm, (**C**), in the CA1 region of hippocampus (CA1, Bregma –1.46 mm, (**D**), in the basolateral amygdala (BLA, Bregma –1.46 mm, (**E**), in the layer II of the piriform cortex (Pir, Bregma –1.46 mm, (**F**) and co-localized with Vglut2 (yellow) in the lateral periaqeductal gray (lPAG, Bregma –4.96 mm, (**G**) and in the raphe magnus nucleus (RMg, Bregma –4.96 mm, (**H**). Scale bar: 20 µm, inset scale bar: 10 µm.

Mouse *Tac4* mRNA expression was investigated using RNAscope in situ hybridization (RNAscope) in various brain regions involved in pain and mood control and even in odor processing. Confocal imaging revealed that *Tac4* mRNA expression was predominantly detected in the *Vglut1/2*-expressing excitatory neurons in most of the brain regions (Fig. 1A,B,C,D,E,F,G,H), including the lateral periaqeductal gray (lPAG, Fig. 1G) and the raphe magnus nucleus (RMg, Fig. 1H), but in addition a few inhibitory interneurons were also *Tac4*^+^ in the prelimbic cortex (Prl, Fig. 1B), in the layer V pyramidal neurons of the primary somatosensory cortex (S1, Fig. 1C), in the CA1 pyramidal cells of the hippocampus (CA1, Fig. 1D), and in the basolateral amygdala (BLA, Fig. 1E), as shown by colocalization with *Gad1* in some cells. Interestingly, *Tac4* expression was found in the olfactory system, both on *Gad1*^+^ GABAergic granule cells and *Vglut1*^+^ principal mitral cells of the olfactory bulb (OB, Fig. 1A), and on the layer II glutamatergic neurons of the piriform cortex, which receive excitatory inputs from the mitral cells (Pir, Fig. 1F)^42^.

### *Tac4* mRNA was expressed in the spinal cord and sensory neurons in the trigeminal and dorsal root ganglia

**Figure 2:**
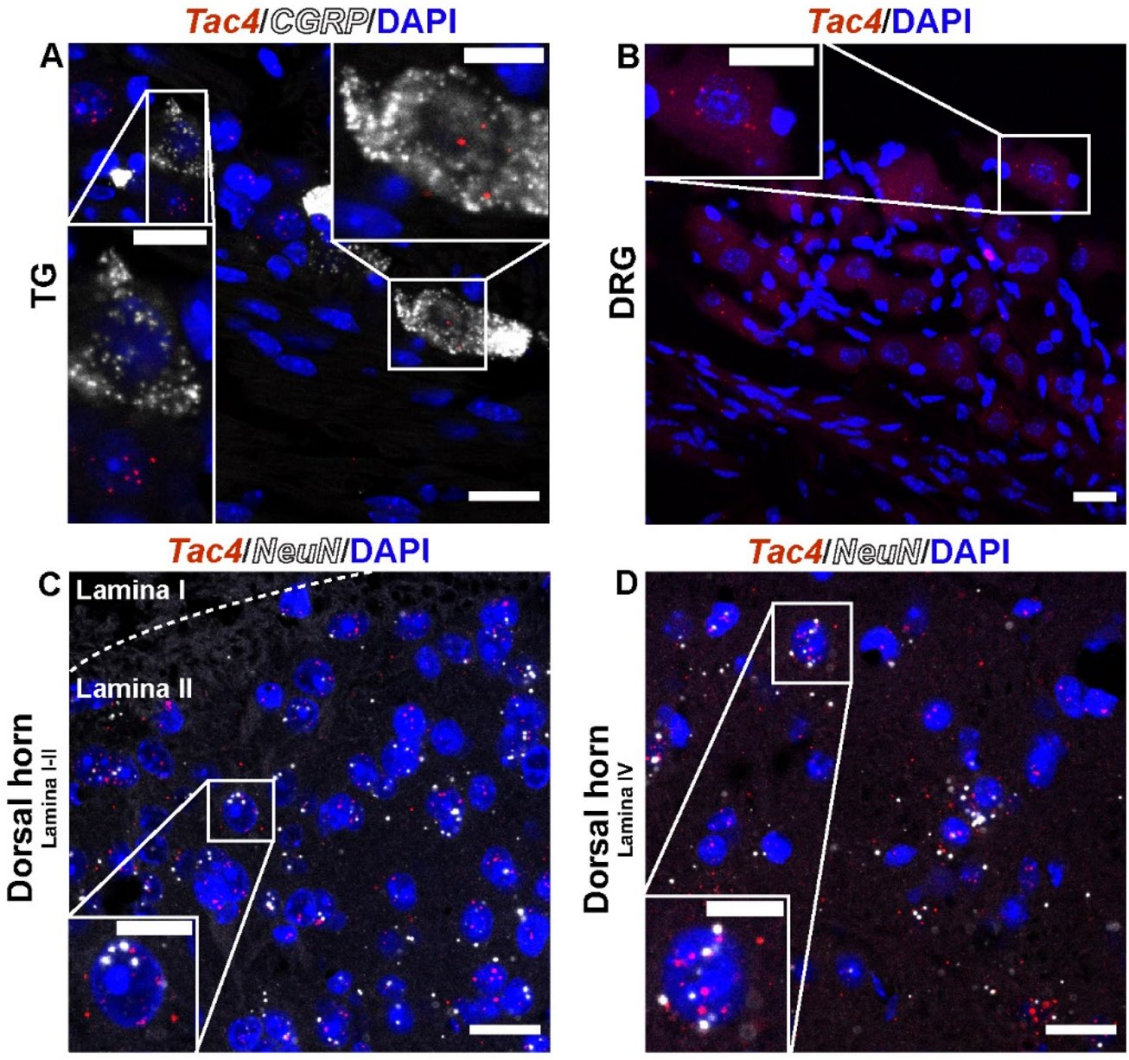
Mouse Tac4 mRNA is expressed in the spinal cord and sensory neurons of trigeminal (TG) and dorsal root ganglia (DRG). Representative confocal images of Tac4 mRNA (red) in TG (**A**), in DRG (**B**) co-localized with (**A**) and without (**B**) calcitonin gene-related peptide (CGRP) mRNA (white). Tac4 was co-expressed with NeuN-positive (white) neurons of the Rexed Lamina II (**C**) and IV (**D**) of the lumbar (L4-L6) dorsal horn of the spinal cord. Scale bar: 20 µm, inset scale bar: 10 µm.

RNAscope revealed that *Tac4* was moderately distributed both in calcitonin gene-related peptide (CGRP)-positive peptidergic and non-peptidergic sensory neurons of mouse trigeminal ganglia (TG, Fig. 2A), as well as in sensory neurons of the dorsal root ganglia (DRG, Fig. 2B). Besides, *Tac4* was found in *NeuN*-expressing neurons of the Rexed Laminae II and IV of the lumbar spinal cord (Fig. 2C, D).

### *Tac4* mRNA was expressed throughout the hypothalamic–pituitary–adrenal axis (HPA) and in the thymus

**Figure 3:**
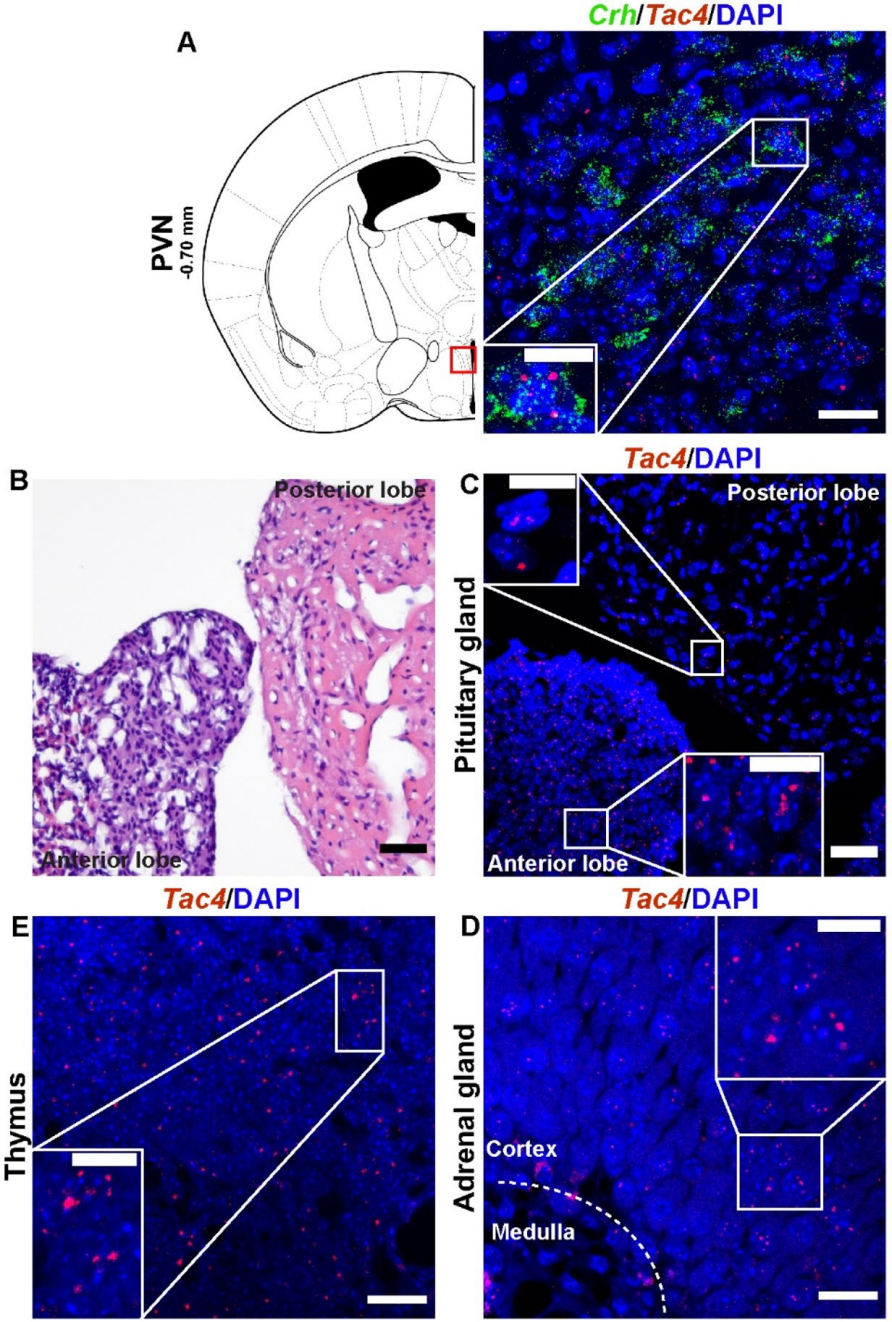
Mouse hypothalamic–pituitary–adrenal (HPA) axis and thymus expressed Tac4 mRNA. Representative confocal images of Tac4 mRNA (red) in the mouse HPA axis co-localized with corticotropin releasing hormone (Crh, green) of the paraventricular nucleus of the hypothalamus (PVN, Bregma –0.70 mm, (**A**), in the posterior and anterior lobe of the pituitary (**C**), in the adrenal cortex (**D**), and in the thymus (**E**). Brightfield microscopy image of the posterior and anterior pituitary gland (**B**). Scale bar: 20 µm, inset scale bar: 10 µm.

Next, we tested whether hemokinin-1 was present throughout the HPA axis as the main effectors of the stress response. *Tac4* mRNA was found in the corticotropin-releasing hormone (*Crh,* green)-expressing cells in the paraventricular nucleus of the hypothalamus (PVN, Fig. 3A), predominantly in the anterior but moderately also in posterior lobe of the pituitary glands (Fig. 3B,C), and mainly in the cortex of the adrenal gland (Fig. 3D). Additionally, *Tac4* mRNA expression was studied in the thymus (Fig. 3E), since in general immune system is one of the primary systems affected by stress and HPA axis activation.

### Stable human *Tac4* expression in the different brain regions

In human tissues, stable *Tac4* gene expression was found in the different brain areas related to nociception and mood regulation. The highest levels could be detected in the frontal, orbitofrontal and prefrontal cortices as well as in the amygdala (Supplementary Figure 3).

### *Tac4* gene deficiency alleviate stress-induced mechanical hyperalgesia but did not influence stress-induced cold allodynia

**Figure 4:**
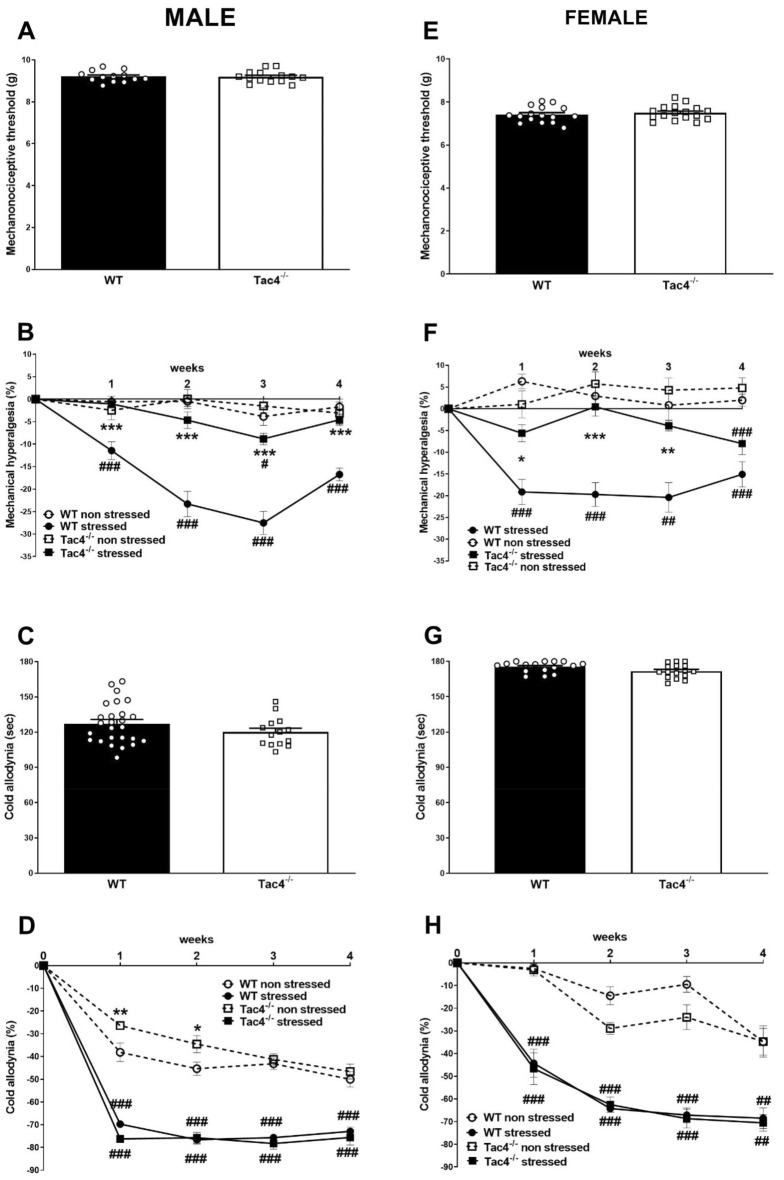
Mechanical hyperalgesia and cold allodynia in male and female WT and Tac4^−/−^ animals. Baseline mechanonociceptive thresholds in grams (**A**, **E**) and cold sensitivity in seconds (**C**, **G**) and their change in % after stress (**B**, **D**, **F**, **H**) during the 4-week-long experimental period. *p < 0.05, **p < 0.01, ***p < 0.001 represents the difference between WT and gene-deleted groups and ^#^*p* < 0.05, ^###^*p* < 0.001 represents the difference between respective non-stressed and stressed groups; for baseline values: *t* test, for changes: two-way analysis of variance (ANOVA) followed by Bonferroni’s post hoc test.

Chronic restraint stress caused significant drop in mechano-nociceptive thresholds in WT animals compared to the non-stressed WT mice. In *Tac4* gene-deleted animals developed no remarkable mechanical hyperalgesia during the whole period of experiment, which resulted in a significant difference compared to the stressed WT animals (Fig. 4B,F). The baseline mechanical threshold did not differ significantly either in male *Tac4*^*−/−*^ (9.193 ± 0.07571 g, *p* = 0.89) and female *Tac4*^*−/−*^ (7.499 ± 0, 0.08038 g, *p* = 0.50) mice compared to their respective WTs (9.209 ± 0.07389 g and 7.415 ± 0.09244 g) (Fig. 4A,E).

Significant cold allodynia developed in all examined groups, which was similar in stressed WT and *Tac*^*−/−*^ groups during the 4-week-long experimental period. Similarly, in non-stressed groups remarkable decrease of cold sensitivity could be measured, while it was significantly smaller in male but not in female *Tac*^*−/−*^ mice during the first 2 weeks compared to the WT animals (Fig. 4D, H). Baseline values were very similar for all *Tac4*^*−/−*^ gene-deleted (120.0 ± 3.334 s, *p* = 0.17, 171.7 ± 1.473 s, *p* = 0.06) and WT (127.3 ± 3.411 s, 175.3 ± 1.140 s) (Fig. 4C, G) male and female groups, respectively.

Mechanical hyperalgesia was also significantly decreased in *Tacr1*^*−/−*^ animals compared to WTs during the whole experiment, but significantly aggravated compared to the non-stressed *Tacr1*^*−/−*^ controls (Supplementary Figure 4B). Significant and similar cold allodynia developed in both *Tacr1*^*−/−*^ and WT animals after stress (Supplementary Figure 4D). The baseline values (mechanical threshold: 9.055 ± 0.08851, *p* = 0.20), cold tolerance: 124.9 ± 3.831 s, *p* = 0.66) of *Tacr1*^*−/−*^ animals did not significantly differ from WTs (Supplementary Figure 4A, C). All statistical data are summarized in Table 1 and Supplementary Table 2.

**Table 1:** Statistical results (*p* values) of the experiments with WT and *Tac4*^*−/−*^ animals. Two-way ANOVA followed by Bonferroni’s posttest in pain measurements and Fischer’s posttest in behavior and organ weight measurements.

	Male				Female			
	WT non-stressed versus WT stressed	*Tac4*^*-/-*^ non-stressed versus *Tac4*^*−/−*^ stressed	WT non-stressed versus *Tac4*^*−/−*^ non-stressed	WT stressed versus *Tac4*^*−/−*^ stressed	WT non-stressed versus WT stressed	*Tac4*^*-/-*^ non-stressed versus *Tac4*^*−/−*^ stressed	WT non-stressed versus *Tac4*^*−/−*^ non-stressed	WT stressed vs. *Tac4*^*−/−*^ stressed
Mechanical hyperalgesia								
1. week	**0.0007**	> 0.9999	> 0.9999	**0.0010**	** < 0.0001**	0.5265	> 0.9999	**0.0124**
2. week	** < 0.0001**	0.7312	> 0.9999	**0.0001**	**0.0001**	0.8236	> 0.9999	**0.0003**
3. week	** < 0.0001**	**0.0219**	> 0.9999	** < 0.0001**	**0.0038**	0.1239	> 0.9999	0.0091
4. week	** < 0.0001**	> 0.9999	> 0.9999	** < 0.0001**	** < 0.0001**	**0.0005**	> 0.9999	0.1589
Cold allodynia								
1. week	** < 0.0001**	** < 0.0001**	0.0998	0.0742	**0.0009**	**0.0009**	> 0.9999	> 0.9999
2. week	** < 0.0001**	** < 0.0001**	0.2671	> 0.9999	** < 0.0001**	** < 0.0001**	0.0618	> 0.9999
3. week	** < 0.0001**	** < 0.0001**	> 0.9999	> 0.9999	** < 0.0001**	**0.0001**	0.2804	> 0.9999
4. week	**0.0001**	**0.0003**	> 0.9999	> 0.9999	**0.0033**	**0.0051**	> 0.9999	> 0.9999
TST								
Immobility time	0.2067	**0.0125**	**0.0368**	0.7035	**0.0020**	**0.0170**	0.2845	0.8510
LDB								
Time spent in the light	0.1951	**0.0369**	**0.0106**	0.1609	**0.0006**	0.0838	0.6194	0.1073
Transitions	0.1177	**0.0016**	**0.0032**	0.4968	**0.0039**	0.8896	0.9009	**0.0030**
OFT								
Time spent in the center zone	0.2072	0.7953	**0.0116**	**0.0361**	0.8187	0.6831	0.6954	0.2991
First enter to the center zone	0.5075	**0.0497**	**0.0010**	0.3329	0.1798	0.7323	0.1631	0.7490
Body elongation	> 0.9999	0.1737	0.1931	**0.0032**	> 0.9999	**0.0211**	> 0.9999	**0.0257**
Organ weights								
Relative adrenal weight	0.1762	0.7689	**0.0074**	**0.0280**	0.1036	0.1671	**0.0030**	**0.0010**
Relative thymus weight	**0.0002**	0.4292	0.1771	0.2563	**0.0076**	** < 0.0001**	0.8120	**0.0157**

Significant alterations are indicated in bold.

### *Tac4* gene deficiency leads to increased vulnerability to stress

**Figure 5:**
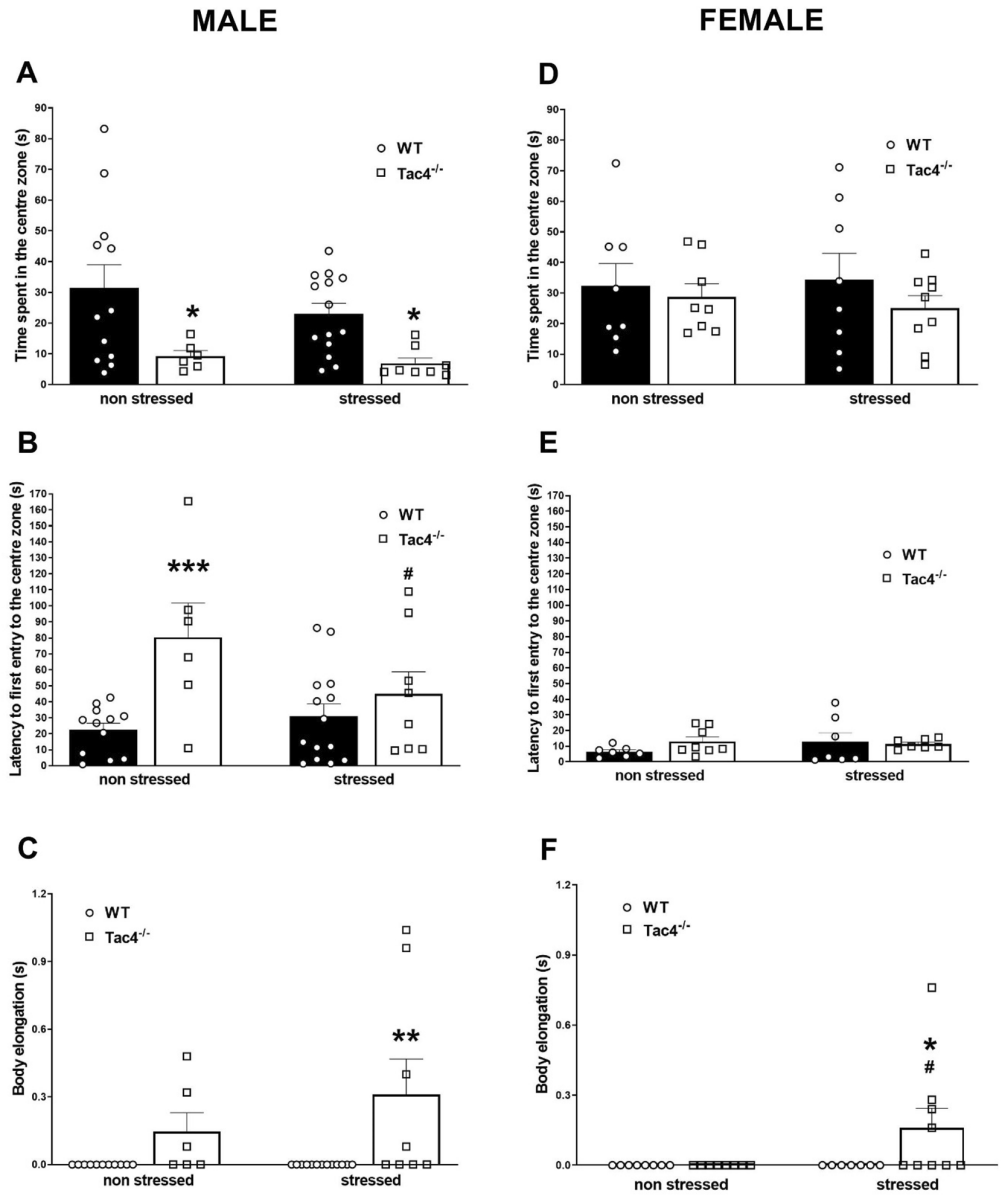
Behavioral changes of WT and Tac4^−/−^ animals. Time spent in the center zone (**A**, **D**), latency to first entry to center zone (**B**, **E**) and body elongation time (**C**, **F**) in open field test at the end of the 4-week-long experimental period. *p < 0.05, **p < 0.01, ***p < 0.001 represents the difference between WT and gene-deleted groups and ^#^*p* < 0.05, represents the difference between respective non-stressed and stressed groups; two-way analysis of variance (ANOVA) followed by Fischer’s post hoc test.

**Figure 6:**
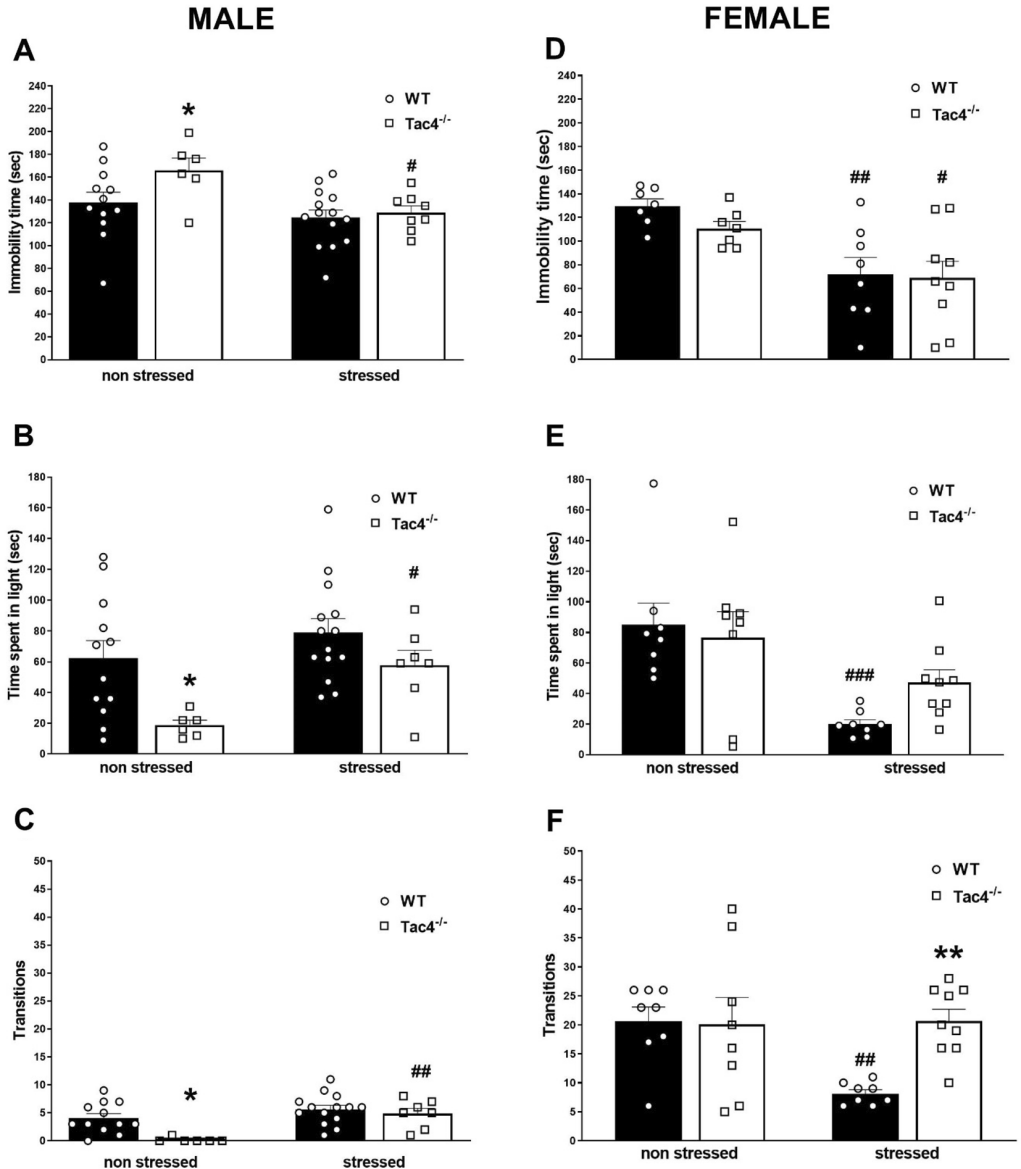
Behavioral changes in WT and Tac4^−/−^ animals. Immobility time in tail suspension test (**A**, **D**), time spent in the lit compartment (**B**, **E**) and transitions between the light and dark compartments in the light–dark box test (**C**, **F**) at the end of the 4-week-long experimental period. *p < 0.05, ***p < 0.001 represents the difference between WT and gene-deleted groups and ^#^*p* < 0.05, ^##^*p* < 0.01, ^##^*p* < 0.001 represents the difference between respective non-stressed and stressed groups; two-way analysis of variance (ANOVA) followed by Fischer’s post hoc test.

In male stressed WT animals no significant change could be measured in parameters of the behavioral tests (TST, LDB, OFT) at the end of week 4 compared to the non-stressed group, while in females immobility time in TST, time spent in the light and number of transitions in LDB significantly decreased. Non-stressed male *Tac4*-deficient mice showed depression-like phenotype, characterized by decreased time spent in the center zone and later entry to the center zone in the OFT (Fig. 5A B), higher immobility time in TST (Fig. 6A), as well as less time spent in light and transitions in LDB (Fig. 6B,C). These differences were absent in female *Tac4*-deficient mice (Fig. 5D,E,F and 6D,E,F). In contrast to the male WTs, immobility time in TST decreased, time spent in the light and number of transitions in LDB increased in stressed *Tac4*^*−/−*^ animals compared to the *Tac4*^*−/−*^ non-stressed male group (Fig. 6A,B,C). Time spent in the central zone was also significantly lower in stressed *Tac4*^*−/−*^ animals, but latency to first entry significantly, body elongation remarkably but not significantly increased in stressed *Tac4*^*−/−*^ mice compared to the non-stressed ones (Fig. 5A,B,C). Interestingly, Tac4 gene-deficient females only showed similar stress-related behavior to males in body elongation and immobility time in TST (Fig. 5F and 6D). Although number of transitions are not remarkably higher in stressed *Tac4*^*−/−*^ females compared to the non-stressed ones, after stress, this value was significantly higher compared to the stressed WT animals (Fig. 6F).

There was no significant difference in non-stressed *Tacr1*^*−/−*^ animals compared to the non-stressed WT group. Due to the stress in *Tacr1*^*−/−*^ mice immobility time in TST and latency to first entry to the central zone of the OFT were significantly decreased compared to the non-stressed *Tacr1*-deficient animals (Supplementary Figure 5A,B,C,D,E,F). All statistical data are summarized in Table 1 and Supplementary Table 2.

### Non-stressed male but not female *Tac4* gene-deficient animals have higher adrenal and lower thymus weights

**Figure 7:**
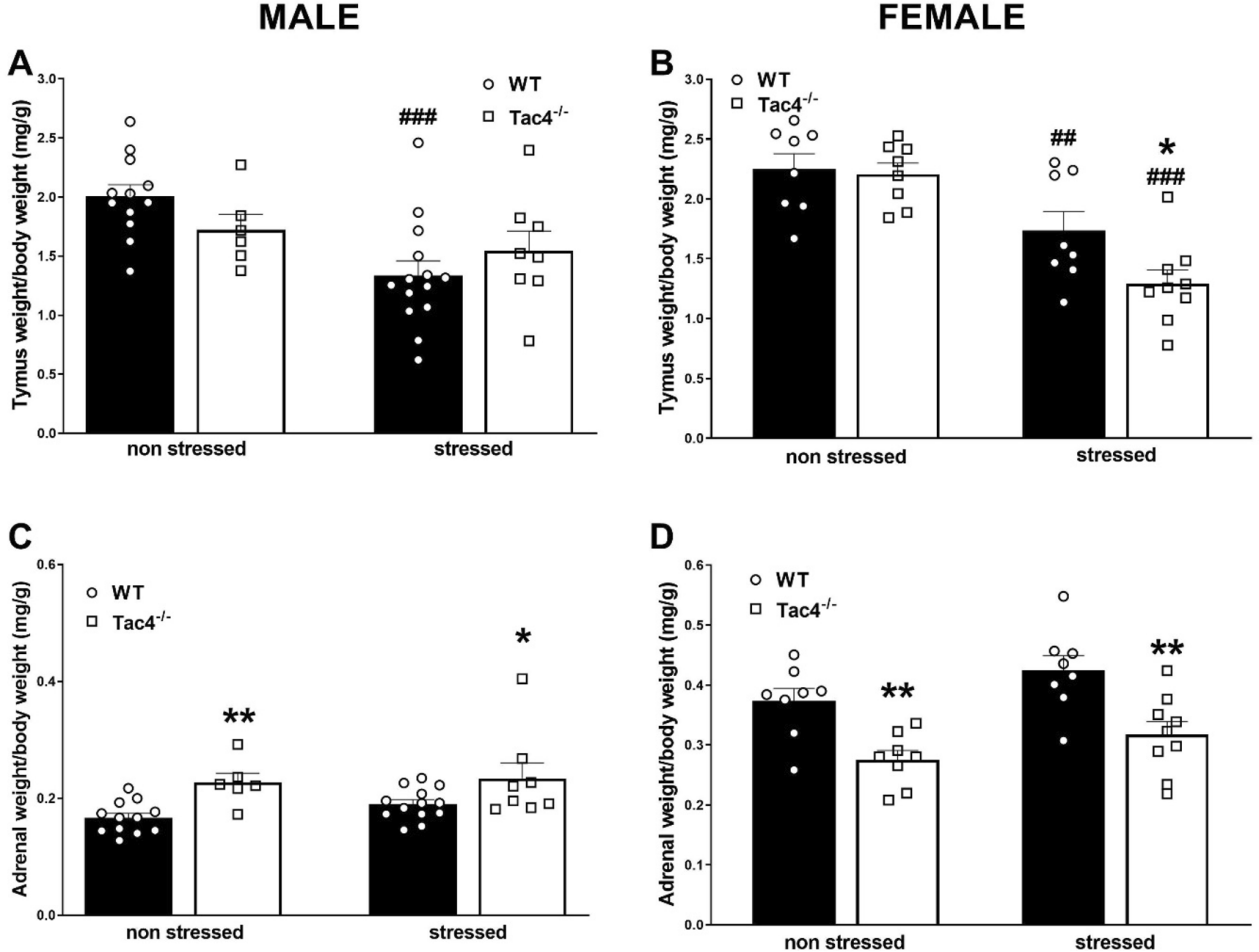
Adrenal gland and thymus weights in WT and Tac4^−/−^ animals. Relative thymus weights (**A**, **B**), as well as relative adrenal gland weights (**C**, **D**) of the animals at the end of the 4-week-long experimental period. *p < 0.05, **p < 0.01 represents the difference between WT and gene-deleted groups and ^##^*p* < 0.01, ^###^*p* < 0.001 represents the difference between respective non-stressed and stressed groups; two-way analysis of variance (ANOVA) followed by Fischer’s post hoc test.

Thymus weights of the WT animals decreased significantly due to the 4-week-long stress (Fig. 7A,B). In contrast, adrenal weights were only slightly, not significantly higher in stressed group compared to the non-stressed controls in both sexes (Fig. 7C,D). In non-stressed male *Tac4*^*−/−*^ animals significantly higher adrenal gland weights and lower thymus weights could be observed, but there was no significant change in stressed group compared to the non-stressed ones (Fig. 7A,C). In stressed female *Tac4-*deficient mice both thymus and adrenal gland weights were significantly lower compared to the stressed WT animals (Fig. 7B,D).

In contrast to *Tac4*^*−/−*^ animals, adrenal weighs of non-stressed *Tacr1*^*−/−*^ mice were lower than the WTs, thymus weights showed no difference compared to the WT counterparts. Interestingly, stressed *Tacr1*^*−/−*^ animals had higher relative adrenal gland as well as thymus weights compared to the stressed WTs (Supplementary Figure 6A,B,C,D). All statistical data are summarized in Table 1 and Supplementary Table 2.

## Discussion

We provide here the first morphological evidence that *Tac4* mRNA encoding mouse HK-1 is expressed in (i) several regions of the central and peripheral nervous systems involved in pain and mood regulation (ii) brain areas related to odor processing, (iii) the main components of the HPA axis, and (iv) the thymus mediating stress responses. Importantly, this is the first study elucidating the neurochemical characteristics of the *Tac4* expressing cells, which demonstrates its expression in of both in excitatory glutamatergic neurons (layer V pyramidal neurons of the primary somatosensory cortex, dorsal hippocampus, principal cells of the hippocampal CA1 region, BLA, piriform cortex) and inhibitory GABAergic interneurons (olfactory bulb, prelimbic cortex) in several brain regions suggesting its potential complex regulatory role in the integration of sensory and stress-related stimuli. Furthermore, regarding pain perception and processing its presence was proven in the peptidergic and non-peptidergic primary sensory neurons as well as secondary sensory neurons in the dorsal spinal horn. These expression patterns well explain our main functional results demonstrating the role of HK-1 in anxiety and stress regulation, as well as chronic stress-induced pain behavior of the mouse. Furthermore, we proved that *TAC4* is also expressed in different human brain regions related to nociception and mood regulation with relatively higher levels in frontal, orbito- and prefrontal cortices, as well as in the amygdala.

The novelty of the present paper is highlighted by the fact that although tachykinines, especially SP have been shown to be abundant in the CNS^43^, but HK-1 distribution has poorly been investigated. So far, HK-1 has been detected in rat primary microglial cultures^44^, and a variety of peripheral immune cells including lymphocytes, monocytes and macrophages^45^. Additionally, mouse *Tac4* mRNA CNS expression assessed by RT-qPCR suggested its presence in the cortex, hippocampus, thalamus, hypothalamus, caudate nucleus, midbrain, brainstem and cerebellum homogenates. Peripherally, *Tac4* mRNA was detected in rodent homogenized TG and DRG samples by RT-qPCR^46^, as well as at cellular level in primary sensory neurons and satellite glial cells of adult mouse and rat trigeminal ganglia using RNAscope^47^. These expression data strongly suggest that HK-1 is a common mediator of several neuronal and immune functions, neuro-immune interactions and neuroinflammatory mechanisms both in the central nervous system and the periphery.

Stress-induced pain involving neuroinflammatory mechanisms and potential autoimmunity^48–50^ is a common feature of several human diseases such as fibromyalgia^51,52^ irritable bowel syndrome and depression. Although there is no optimal model which could mimic all aspects and symptoms of fibromyalgia which is related to currently unraveled complex etiology and pathophysiology, several models are used to reflect different characteristics and mechanisms of the disease^53^ . The present chronic restraint model is suitable for the investigation of pain-related behavior exclusively induced by psychological stimuli. We previously described that the daily 6-h-long restraint induces significant reduction in both the mechanonociceptive threshold and cold tolerance in mice^14^. This was reproducible in the present experimental series supporting the relevance of this paradigm to investigate stress-induced pain mechanisms. We demonstrate here that mechanical hyperalgesia as the main outcome parameter of this study is absent in case of HK-1 deficiency in both sexes supported by several data suggesting a mediator role of HK-1 in pain conditions of different origin^18^. However, the molecular mechanism of action and the involvement of the NK1 tachykinin receptor varies between the different animal models depending on the pathophysiological processes^19–21^. The present results show that pronociceptive action of HK-1 is, at least partially, mediated by the NK1 receptor, since both the lack of HK-1 and NK1 lead to significantly attenuated mechanical hyperalgesia. In HK-1 deficient mice this effect was more pronounced, since the mechanical threshold is almost the same as in the non-stressed group, while 10–15% mechanical hyperalgesia was measured in NK1-deficient animals. Since cold allodynia mainly mediated by peripheral mechanisms was not influenced in any stressed groups, we suppose that the role of HK-1 in pain sensitization is—at least partially—centrally mediated.

In accordance with the nociceptive results, behavioral alterations related to spontaneous locomotor activity, anxiety and mood regulation are mild or absent in male WT mice at the end of the 4-week investigation period. This is likely to be due to the adaptation to the same type of chronic stress, which is well established in this experimental paradigm^54,55^. In contrast, in female WT mice significant changes in time spent in light and transitions in LDB as well as immobility time in TST were found in this experimental paradigm, highlighting remarkable sex-related differences in stress sensitivity and adaptation involving complex peripheral and central neuroendocrine processes. Adaptation is also confirmed by the lack of difference between the adrenal gland weights in either male or female WT mice. However, thymus weight decrease can be observed even after 4 weeks in both sexes which seems to be a more sensitive and longer-lasting indicator of chronic stress^56^. Higher baseline anxiety and depression-like behaviors were detected in male but not in female HK-1-deficient mice, while other parameter (e.g. body elongation increase) altered by stress was present in both male and female HK-1 deficient animals. This demonstrates strong interactions between HK-1, neurotransmitters, sex and stress hormones at different levels highlighting the importance of investigating both sexes in preclinical studies.

Our previous results proved that HK-1 and NK1 are involved differently in acute stress reactions, HK-1 reduces, NK1 induces anxiety and depression-like behavior^22^. The same tendency could be observed in this series of experiments for the NK1 receptor, but the difference was not significant in the gene-deleted group. This could be explained by the differences of the pathophysiological mechanisms in the models: the previously published acute tests were performed on naive animals without any intervention, while the present non-stressed group was measured many times to register the nociceptive thresholds and weights during the 4-week-long experimental period which can influence the behavioral results. In accordance with previous data, the lack of NK1 receptor did not cause significantly altered behavior compared to WT animals but promotes stress-adaptation.

Several sensory cues are processed by the olfactory bulb, which directly and indirectly projects to the piriform and prefrontal cortices, amygdala, hypothalamus, and hippocampus^57,58^. Thus, the limbic system is prominently influenced by olfactory inputs and therefore HK-1 might have a role in these functions. The hippocampus, which shows abundant expression of the *Tac4* gene, is the key regulator of stress responses. It negatively influences the HPA axis and the release of glucocorticoid stress hormones^59^ supporting our previous^22^ and present conclusion for HK-1 being a stress and anxiety reducing factor. Moreover, the hippocampus is shown to be highly vulnerable to elevated glucocorticoid level, which is associated with stress-induced hippocampal atrophy^60,61^. Thus, reciprocal connection between the hippocampus and HPA axis/stress hormones have been well-investigated. Our findings regarding higher stress levels in non-stressed *Tac4* deficient mice as shown by the higher adrenal gland and lower thymus weights and earlier behavioral data^22^ together with lower stress-induced pain responses might be explained by the glucocorticoid-mediated analgesic actions^62,63^. Recent data provided evidence that HK-1 in carp pituitary cells up- and downregulate the expression of several important molecules involved in mood regulation and stress responses (cocaine- and amphetamine-regulated transcript, somatostatin receptors, growth factors)^64^.

Besides the adaptation process in the experimental paradigm, another limitation of the present study is that we detected *Tac4* mRNA expression which might not necessarily reflect HK-1 production. However, due to the lack of specific antibody being appropriate to differentiate from SP by immunohistochemistry, it cannot be detected at the protein level. The main advantage of the applied highly sensitive RNAscope technology is, that ensures good resolution at single mRNA molecule level preserving the morphological context^65^.

The main novelty of the present study is that this is the first description and neurochemical characterization of HK-1 expression pattern in pain and stress-related brain regions and peripheral tissues. This expression data supports our functional results concluding on the mediator role of HK-1 in chronic stress-induced pain and stress regulation both in the central nervous system and the periphery. The tachykinin NK1 receptor might have a role in mediating the pain-producing, but not the anti-anxiety and antidepressive actions of HK-1. These distinct mechanisms of action might provide potential therapeutic perspectives for stress-induced pain conditions such as fibromyalgia.

### Supplementary Information

Supplementary Information.

## Data Availability

The datasets used and/or analyzed during the current study available from the corresponding author on reasonable request.

## Abbreviations

BL: Basolateral amygdaloid nucleus;
CA1: Field CA1 of hippocampus;
CGRP: Calcitonin gene-related peptide;
*Crh*: Mouse corticotropin-releasing hormone encoding gene;
CRS: Chronic restraint stress;
DAPI: 4′,6-Diamidino-2-phenylindole;
DPA: Dynamic plantar aesthesiometer;
DRG: Dorsal root ganglion;
FST: Forced swim test;
*Gad1*: Mouse glutamate decarboxylase 1 encoding gene;
HK-1: Hemokinin-1;
HPA: Hypothalamic–pituitary–adrenal;
LDB: Light–dark box test;
NK-1: Neurokinin 1;
OB: Olfactory bulb;
OFT: Open field test;
Pir ctx: Piriform cortex;
*Ppib*: Mouse peptidyl-prolyl cis–trans isomerase B encoding gene;
*Polr2a*: Mouse DNA-directed RNA polymerase II subunit RPB1 encoding gene;
PVN: Paraventricular nucleus of the hypothalamus;
S1: Primary somatosensory cortex;
*Tac4*: Mouse hemokinin-1 encoding gene;
*Tac1r*: Mouse tachykinin NK1 receptor encoding gene;
TG: Trigeminal ganglion;
TST: Tail suspension test;
*Ubc*: Mouse ubiquitin C encoding gene;
*Vglut1*: Mouse vesicular glutamate transporter 1 encoding gene (*Slc17a7*);
*Vglut2*: Mouse vesicular glutamate transporter 2 encoding gene (*Slc17a6*);
WT: Wildtype
