# Supplementary material for: Hemokinin-1 is a mediator of chronic restraint stress-induced pain
Source: Sci Rep. 2023 Nov 16;13:20030. doi: 10.1038/s41598-023-46402-7 (PMC10654722; doi:10.1038/s41598-023-46402-7)
Supplement: Supplementary file 1 — Supplementary Information. [file 41598_2023_46402_MOESM1_ESM.docx]

**
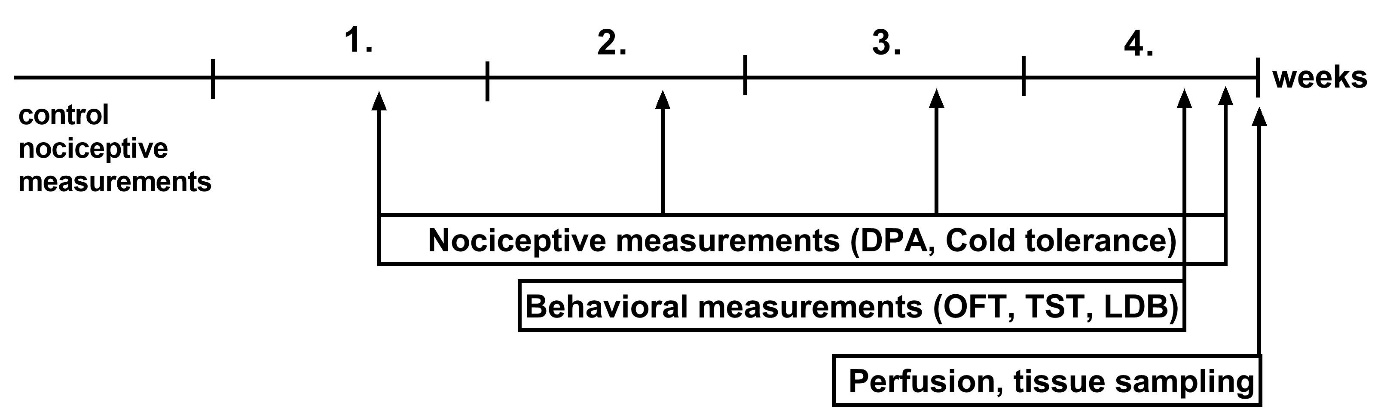
Suppl. Fig. 1.** Experimental protocol of chronic restraint stress, nociceptive and behavioral tests and perfusion. DPA, dynamic plantar aesthesiometry; OFT, open-field test; TST, tail suspension test; LDB, light–dark box test.


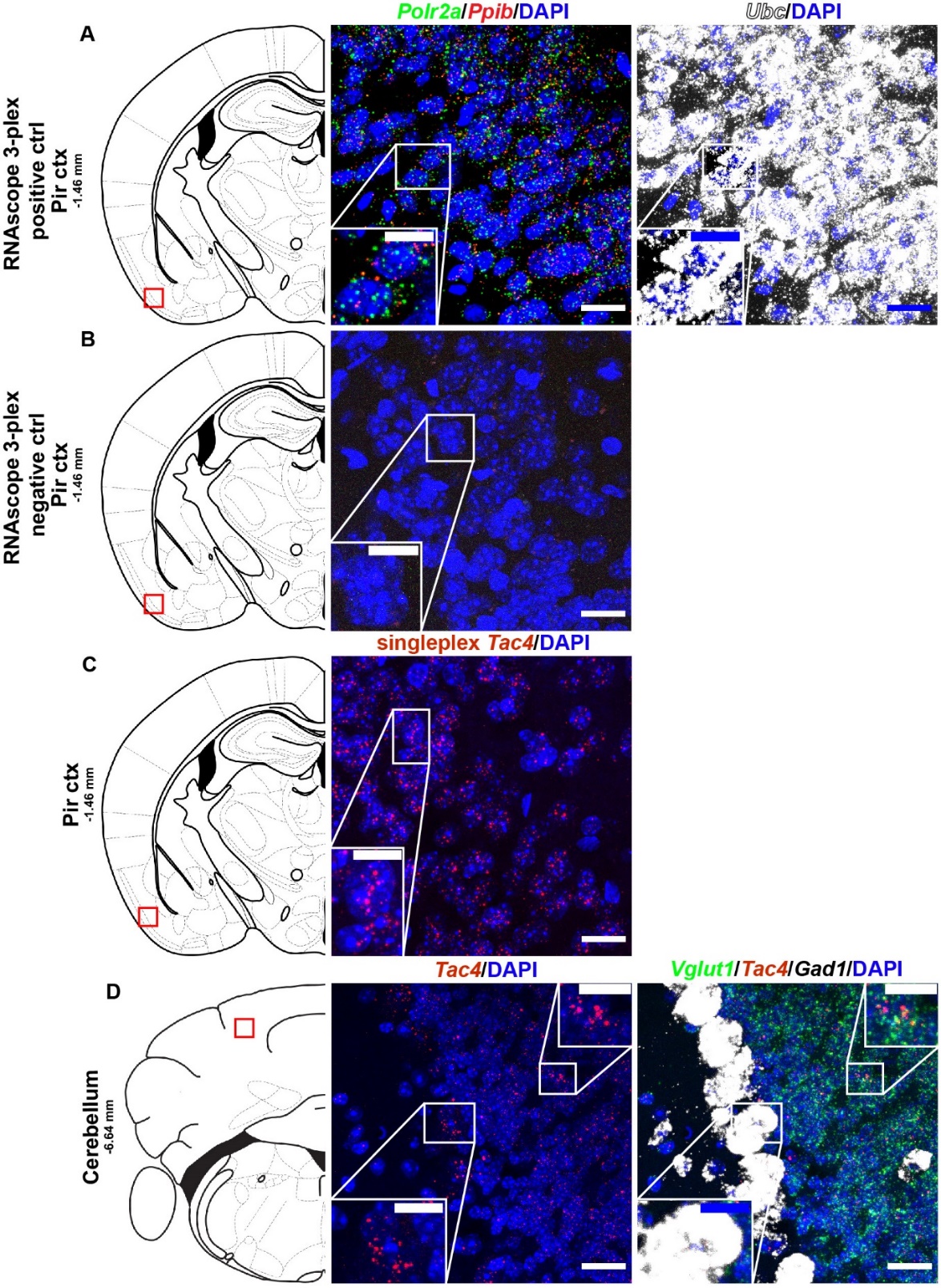


**Suppl. Fig. 2.** Representative technical control conditions for the RNAscope studies. RNAscope 3-plex positive control probes specific to mouse low copy Polr2a (green), medium copy Ppib (red) and high copy Ubc (white) mRNA (A), RNAscope 3-plex negative control specific to bacterial dapB gene (B) and singleplex Tac4 condition (red, C) depicted in the piriform cortex (Pir ctx, Bregma –1.46 mm). Tac4 mRNA expression co-localized with Vglut1 and Gad1 mRNA imaged in mouse cerebellum, served as positive brain tissue control (Bregma –6.64 mm, D). N=1, scale bar: 20 µm, inset scale bar: 10 µm.

**Suppl. Fig. 3.** Relative human TAC4 mRNA of all (A), male (B) and female (C) patients measured by RT-qPCR in different brain regions from microdissected post-mortem tissues (N=4–12). The geometric mean of the reference gene Ct values was determined, and SEM of Tac4 mRNA expression relative to the reference genes (human POLR2A, PES1 and IPO8) was calculated by the 2-ΔCt formula to compare distinct brain areas and represented as scattered dot plots.

| **Patient No.** | **Age (years)** | **Cause of death (and chronic disease)** |
| --- | --- | --- |
| **Male** | | |
| **1** | **42** | **cardiac insufficiency** |
| **3** | **66** | **cardio-pulmonal insufficiency (leukemia)** |
| **5** | **52** | **cardiac insufficiency** |
| **7** | **53** | **pulmonal insufficiency** |
| **8** | **63** | **pulmonal insufficiency** |
| **9** | **61** | **cardiac insufficiency (diabetes)** |
| **10** | **27** | **pulmonal insufficiency** |
| **12** | **50** | **stroke** |
| **13** | **55** | **cardio-pulmonal insufficiency** |
| **14** | **68** | **cardiac insufficiency** |
| **Female** | | |
| **2** | **79** | **cardiac insufficiency** |
| **4** | **48** | **cardiac insufficiency** |
| **6** | **44** | **cardiac insufficiency** |
| **11** | **26** | **cardiac insufficiency** |
| **15** | **72** | **cardio-pulmonal insufficiency** |

**Suppl. Table 1.** Medical data of human subjects (post-mortem samples)

**Suppl. Fig. 4.** Mechanical hyperalgesia and cold allodynia in WT and Tacr1^-/-^ animals. Baseline mechano-nociceptive thresholds in grams (A) and cold sensitivity in seconds (C) and their change in % after stress (B, D) during the 4-week-long experimental period. *p<0.05, **p<0.01, ***p<0.001 represents the difference between WT and gene-deleted groups and ###p<0.001 represents the difference between respective non-stressed and stressed groups; two-way analysis of variance (ANOVA) followed by Fischer’s post hoc test.


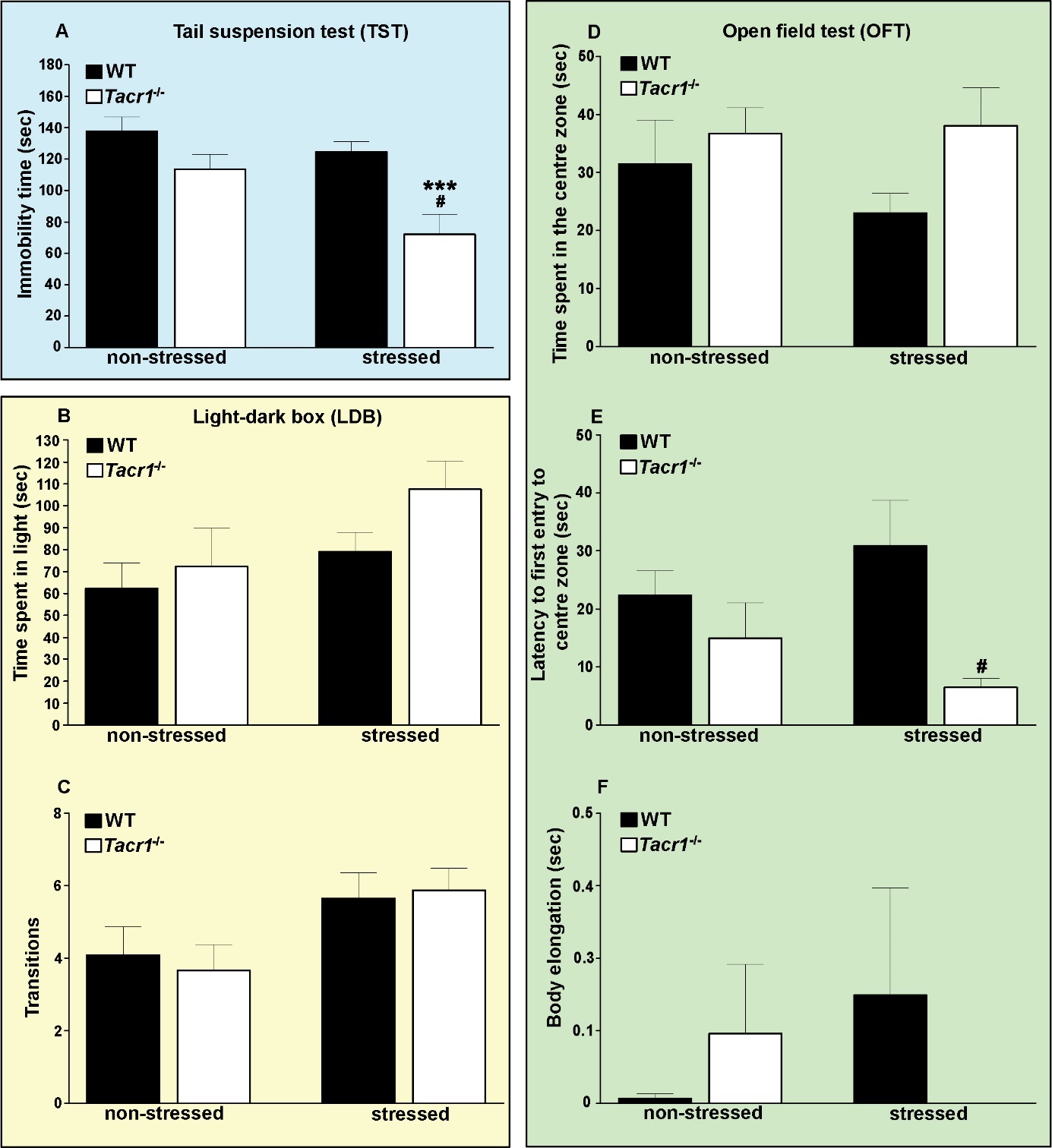
**Suppl. Fig. 5.** Behavioral changes in WT and Tacr1^-/-^ animals. Immobility time in tail suspension test (A), time spent in the lit compartment (B) and transitions between the light and dark compartments in the light-dark box test (C) as well as time spent in the center zone (D), latency to first entry to center zone (E) and body elongation time in open field test (F) at the end of the 4-week-long experimental period. ***p<0.001 represents the difference between WT and gene-deleted groups and #p<0.05 represents the difference between respective non-stressed and stressed groups; two-way analysis of variance (ANOVA) followed by Fischer’s post hoc test.


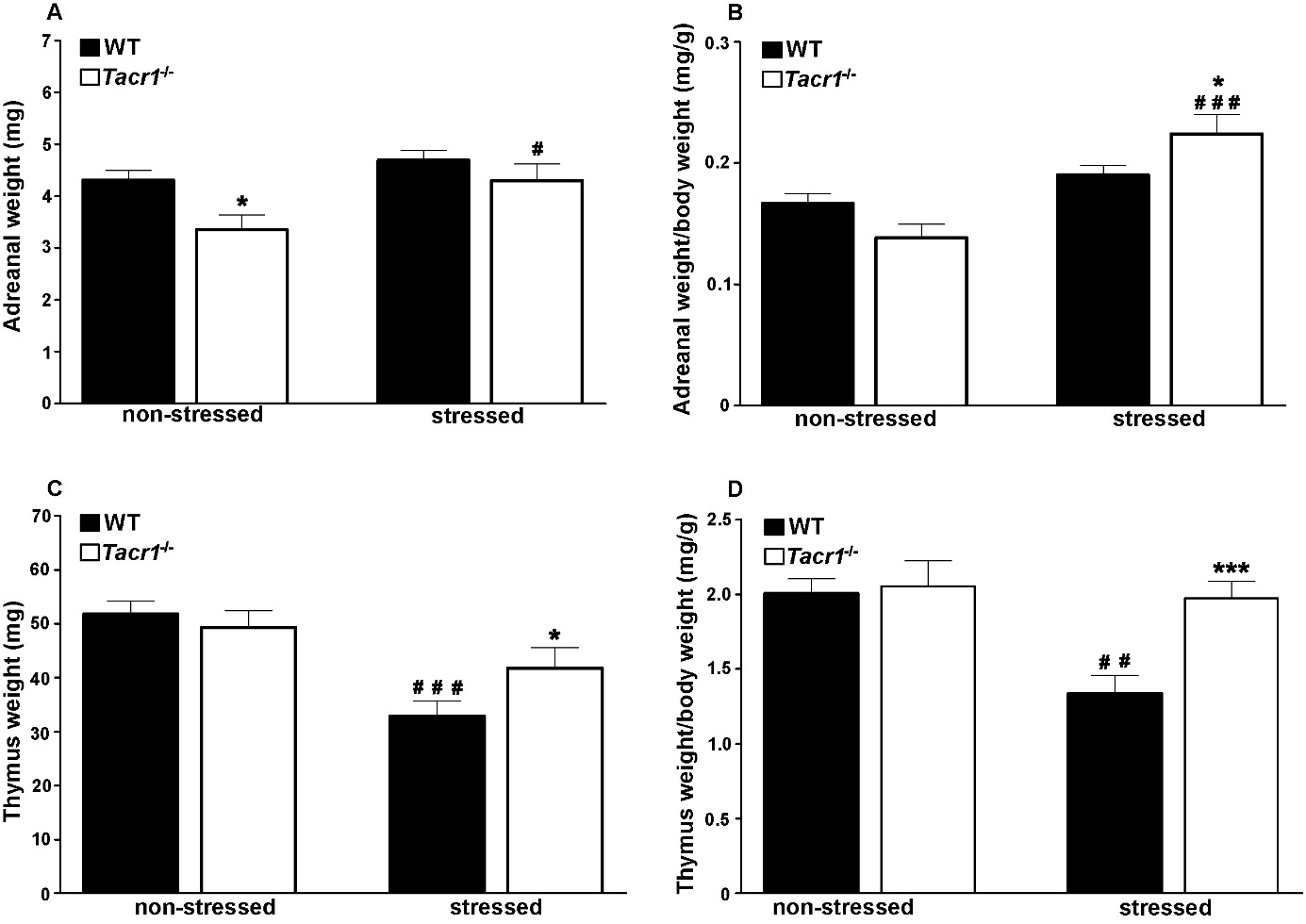
**Suppl. Fig. 6.** Adrenal gland and thymus weights in WT and Tacr1^-/-^ animals. Absolute (A), and relative adrenal gland weights (B) as well as absolute (C) and relative thymus weights (D) of the animals at the end of the 4-week-long experimental period. *p<0.05, ***p<0.001 represents the difference between WT and gene-deleted groups and #p<0.05, ##p<0.01, ###p<0.001 represents the difference between respective non-stressed and stressed groups; two-way analysis of variance (ANOVA) followed by Fischer’s post hoc test.

|  | | WT non-stressed vs. WT stressed | Tacr1^-/-^ non-stressed vs.  Tacr1^-/-^ stressed | WT non-stressed vs. Tacr1^-/-^ non-stressed | WT stressed vs.  Tacr1^-/-^ stressed |
| --- | --- | --- | --- | --- | --- |
| Mechanical hyperalgesia | 1. week | **0,0007** | >0,9999 | >0,9999 | 0,1060 |
|  | 2. week | **<0,0001** | **0,0369** | >0,9999 | **0,0446** |
|  | 3. week | **<0,0001** | **0,0048** | 0,0848 | **0,0021** |
|  | 4. week | **<0,0001** | >0,9999 | >0,9999 | 0,2999 |
| Cold allodynia | 1. week | **<0,0001** | **<0,0001** | 0,6595 | >0,9999 |
|  | 2. week | **<0,0001** | **0,0039** | 0,5484 | >0,9999 |
|  | 3. week | **<0,0001** | **0,0068** | >0,9999 | 0,7380 |
|  | 4. week | **0,0001** | **0,0023** | 0,9889 | >0,9999 |
| TST | immobility time | 0,2614 | **0,0270** | 0,1612 | **0,0003** |
| LDB | Time spent in the light | 0,2635 | 0,0896 | 0,5967 | 0,0940 |
|  | Transitions | 0,1097 | 0,0994 | 0,7323 | 0,8297 |
| OFT | Time spent in the center zone | 0,2630 | 0,9001 | 0,5761 | 0,0803 |
|  | First enter to the center zone | 0,2916 | 0,4410 | 0,4722 | **0,0100** |
|  | Body elongation | 0,3036 | 0,6102 | 0,6083 | 0,3385 |
| Adrenal weight | Absolute | 0,1915 | **0,0198** | **0,0117** | 0,2335 |
|  | Relative | 0,0708 | **<0,0001** | 0,0811 | **0,0226** |
| Thymus weight | Absolute | **<0,0001** | 0,1505 | 0,6009 | **0,0422** |
|  | Relative | **0,0001** | 0,7096 | 0,8033 | **0,0013** |

**Suppl. Table 2.** Statistical results (p values) of the experiments with WT and *Tacr1^-/-^* animals. Two-way ANOVA followed by Bonferroni’s posttest in pain measurements and Fischer’s posttest in behavior and organ weight measurements. Significant alterations are indicated in bold.
